# Incorporation of Yogurt Acid Whey in Low-Lactose Yogurt Ice Cream

**DOI:** 10.3390/foods12203860

**Published:** 2023-10-21

**Authors:** Lambros Sakkas, Marianna Karela, Evangelia Zoidou, Golfo Moatsou, Ekaterini Moschopoulou

**Affiliations:** Laboratory of Dairy Research, Department of Food Science and Human Nutrition, Agricultural University of Athens, Iera Odos 75, 118 55 Athens, Greece; lasakkas@hotmail.com (L.S.); marianna_kar@hotmail.com (M.K.); ezoidou@aua.gr (E.Z.); mg@aua.gr (G.M.)

**Keywords:** ice cream, yogurt, acid whey, lactose, circular economy

## Abstract

Yogurt acid whey (YAW), a by-product of strained yogurt production, is a strong environmental pollutant because of its high organic load. Hence, efforts are made for its utilization to minimize its disposal in the environment. This study deals with the incorporation of YAW in yogurt ice cream (YIC) by partial replacement of yogurt with simultaneous lactose hydrolysis (LH) of the formulated YIC mix. Six YIC mix formulations were made, two without YAW (non-LH- and LH-control samples, A and AH), two with 12.5% YAW (samples B and BH), and two with 18.75% YAW (samples C and CH). The results showed that the partial replacement of yogurt with YAW decreased significantly (*p* < 0.05) the total solids of B, BH, C, and CH products (31.72 ± 0.14%, 31.92 ± 0.21%, 30.94 ± 0.14%, and 31.27 ± 0.10%, respectively) compared to the total solids of control products A and AH (33.30 ± 0.36% and 33.74 ± 0.06%, respectively). In contrast, the overruns increased (51.50 ± 2.36%, 58.26 ± 0.09%, 56.86 ± 1.92%, and 65.52 ± 1.30% for the B, BH, C, and CH products, respectively) compared to control samples (42.02 ± 2.62% and 49.53 ± 2.12% for A and AH, respectively). LH significantly decreased the freezing point and the viscosity of the YIC mixes but increased the overruns of the products as shown previously. YAW significantly decreased the hardness of the B and C products (56.30 ± 2.11 N and 43.43 ± 3.91 N, respectively) compared to control A (81.14 ± 9.34 N), and LH decreased it even more, leading to a rather soft scoop YIC. AH, BH, and CH YICs exhibited better melting properties despite the lack of fat destabilization in all samples. After 60 days of storage, counts of yogurt starter microorganisms were still >10^7^ cfu/g and DPPH radical scavenging activity had increased in all products. In the sensory evaluation test, lactose-hydrolyzed samples AH, BH, and CH had less intense sandiness and, as expected, more intense sweetness. In conclusion, in the framework of the circular economy, it is possible for the YAW to be used as a resource material at a ratio of 12.5% to produce a YIC product without leaving behind any new waste.

## 1. Introduction

Acid whey (AW) is a by-product generated from Greek yogurt (yogurt acid whey, YAW), cottage cheese, and different milk permeates. However, the highest quantity of AW worldwide comes from the continuous increase in production of Greek yogurt because of its high nutritional value. To produce 1 kg of Greek yogurt, approximately 3 kg of YAW are discarded [1]. YAW has a pH of 3.5–4.5 and contains about 5–7% total solids, of which the main constituent is lactose, i.e., 3.37–4.99% [2], and because of this it is a strong pollutant having a high average BOD value of 40,000 mg/L [3]. Furthermore, the presence of lactic acid and minerals in YAW can lead to issues such as powder stickiness during the drying process. Therefore, research is being carried out to remove the mineral content using nanofiltration and electrodialysis [4,5], to improve the quality of the produced powders. Moreover, studies have investigated the use of YAW in other applications, such as meat marination and dry curing [6,7], and the production of monosaccharides and minerals [8]. However, a quantity of the YAW that was used was left behind as new waste, albeit with fewer negative environmental impacts. On the other hand, the application of YAW on land as fertilizer [9] as well as its incorporation in foods, e.g., sauces [10], is a good practice that uses the YAW without leaving any waste.

Yogurt ice cream (YIC), also known as frozen yogurt, is a frozen dessert that is made with yogurt, i.e., milk fermented with the use of the bacteria *Lactobacillus delbrueckii* ssp. *bulgaricus* and *Streptococcus thermophilus*, and may contain sweeteners, flavors, colorants, stabilizers, and emulsifiers. It should have a minimum of 0.3% titratable acidity, expressed as lactic acid, and it must contain a minimum of 2.7% protein, less than 10% fat, and a minimum 10^7^ cfu/g sum of microorganisms constituting the starter culture [11]. In most countries, however, there are no standards for YIC and hence many products exist in which the acidity and the yogurt flavor is from the addition of citric acid and yogurt flavors or yogurt powders. Commercial YICs sold in the USA market contain 1.7–5.9% fat, 1.6–3.8% protein, 0.7–1.1% ash, and 28.8–34.2% total solids [12,13]. Regarding the manufacturing process, YIC is made by mixing ice cream mix base and plain yogurt at ratios of 70–80% and 30–20%, respectively, following the same stages of technology as in any type of ice cream, i.e., YIC mix aging, freezing, packaging, hardening, and storage at −18 °C to −20 °C. This is an indirect method for the acidification of the product, while fermenting all ingredients with yogurt starter cultures, cooling and freezing after incubation is the direct method [14].

In general, for ice cream products, the ice cream mix formulations are made using either whole/skimmed milk or a mixture of milk and water as the liquid for the dilution of the solid materials, i.e., sugars, emulsifiers/stabilizers, powders, etc. Therefore, YAW could be part of a formulation for YIC production without dramatically changing the final taste since it is a constituent of yogurt. However, the incorporation of YAW in the ice cream mix lowers the pH and consequently limits the heating of the ice cream mix at the minimum pasteurization temperatures for ice cream mixes, i.e., 83 °C for 15 s (HTST method) or at 69 °C for 30 min (LTLT method) [12], due to the possible precipitation of casein. Another possible problem regarding the incorporation of YAW in ice cream is sandiness, a texture fault that arises due to lactose crystallization. This problem can be solved by lactose hydrolysis to glucose and galactose, which inhibits lactose crystallization and hence ice cream sandiness. In parallel, lactose hydrolysis helps consumers who suffer from lactose intolerance, since the monosaccharides glucose and galactose are readily absorbed in the small intestine and prevent the occurrence of symptoms such as abdominal pain, flatulence, and diarrhea [15,16,17,18]. The definitions, however, for lactose-free, zero-lactose, lactose-reduced, and low-lactose dairy products vary from country to country. The threshold levels for lactose content in some European countries range from 0 to 100 mg/100 g for lactose-free products and from 0 to 1 g/100 g for low-lactose products [19].

In literature, most of the studies concern YIC fortified with probiotics, different flavors, syrups, and dietary fibers [20,21,22,23,24,25,26,27], or the addition of fat replacers and different stabilizers [28,29,30]. To the best of our knowledge, except for the study by Silva and Bolini [31], who used powdered acid whey from cheese or casein production to produce ice cream, no other study has used liquid acid whey or YAW in ice cream manufacture. Liquid acid whey or YAW are just pasteurized and not over-processed, as with powdered AWs, and can be readily used in some products in the framework of the circular economy. The aim of this research was to produce a novel YIC product by replacing part of the yogurt with YAW in the YIC mix formulation.

## 2. Materials and Methods

### 2.1. Materials

For the ice cream mix, homogenized full-fat bovine milk (3.5% fat), homogenized fresh cream (35% fat), and sucrose were obtained from the retail market. Moreover, medium heat bovine skimmed milk powder (SMP, Arla Foods, Visby, Sweden) with 1.25% fat, 35% protein, 52% lactose, and 8% ash, and the commercial blend of emulsifiers and stabilizers (E/S) Cremodan SE 334 VEG (mono- and diglycerides of fatty acids, guar gum, cellulose gum and carrageenan, DuPont, Wilmington, DE, USA) were used. Bovine strained yogurt with 2% fat, 4% lactose, 9% protein (14% MSNF), and raw YAW was provided by the Greek dairy company OLYMPOS. The YAW had pH 4.5 and contained 5.71% total solids, 3.57% sugars, 0.26% protein, 0.07% fat, 0.89% ash, 0.124% calcium, 0.015% magnesium, 0.152% potassium, and 0.052% sodium. For lactose hydrolysis, a β-galactosidase (NOLA™ Fit 5500, HANSEN, Melbourne, Australia) which can act at lower pH values, i.e., with an optimum pH of 5–7, was used.

### 2.2. Ice Cream Mix Formulation

Six YIC mixes were formulated to contain 12–14% MSNF and 30–34% total solids, as shown in Table 1. In control samples A and AH, without YAW, 75% ice cream mix and 25% strained yogurt were mixed. In samples B and BH, YAW replaced half of the yogurt, i.e., 75% ice cream mix, 12.5% strained yogurt, and 12.5% YAW were mixed, and in samples C and CH, YAW replaced three-quarters of the yogurt, i.e., 75% ice cream mix, 6.5% strained yogurt, and 18.75% YAW were mixed. In samples AH, BH, and CH, lactose was hydrolyzed.

### 2.3. Ice Cream Production

Each YIC mix base was prepared by mixing milk, fresh cream, E/S blend, and sucrose, heated at 82 °C for 1 min under continuous agitation, cooled down to 25 °C, and finally kept at 4 °C for 4 h. Moreover, raw YAW was pasteurized at 72 °C for 1 min and cooled down to 4 °C. After cooling, the YIC mix base was mixed with strained yogurt and YAW at different ratios as shown in Table 1. To AH, BH, and CH YIC mixes, the enzyme β-galactosidase was added at a ratio of 1% and then the six YIC mixes remained at 4 °C for 16 h to age. Freezing of each YIC mix took place in random order using a domestic vertical ice cream freezer with a 1.5 L capacity (Arktic Hendi B.V., Ede, The Netherlands). Freezing lasted for 45 min and the temperature of the YIC at the end was −5 °C. About 0.8 kg of YIC was produced per batch, which was then packed in 100 and 150 mL sterilized plastic cups, and finally placed at −22 °C for hardening and storage. The experiment was carried out in triplicate in three successive weeks.

### 2.4. Analyses of YIC Mix Samples

pH was directly measured using a digital pH meter (HI99161, HANNA INSTRUMENTS, Smithfield, RI, USA), and acidity, expressed as (%) lactic acid, was determined by titration using NaOH N/9 solution.

Fat, protein, and total solids contents were determined using Milkoscan FT-120 (Foss, Hilleroed, Denmark) after dilution with ultra-pure water at a ratio of 1:3. Ash content was determined by heating the YIC mix at 550 °C for 6 h. Calcium, magnesium, potassium, and sodium contents were determined using the Atomic Absorption Spectrometry method of the International Dairy Federation [32] on a Shimadzu AA-6800 Atomic Absorption Spectrophotometer equipped with the autosampler Shimadzu ASC-6100 and the software WizAArd v. 2.30 (Shimadzu Corporation, Kyoto, Japan). Lactose content was determined by the HPLC method on a Perkin Elmer Flexar system (Shelton, CT, USA), according to the method described by Karastamatis et al. [2] using 10 g of sample in the sample preparation.

The freezing point was determined using a cryoscope (CryoStar 1, Funke Gerber, Berlin, Germany) after diluting one part YIC mix with three parts ultra-pure water [12,17].

The water activity (a_w_) of the YIC mix was measured at 22 ± 1 °C on the instrument AQUALAB Dew Point Water Activity Meter (Aqualab, METER Group, Inc., Pullman, WA, USA).

Viscosity (mPa·s) was determined at 50 rpm at room temperature (20 ± 1 °C) using a Viscolead one rotational viscometer (Fungilab S/A, Barcelona, Spain) equipped with spindle No. L2.

All analyses of the YIC mix were performed in triplicate.

### 2.5. Analyses of YIC Samples

Overrun (%) of the YIC samples was calculated based on weights of a specific volume of ice cream mix and ice cream according to Goff and Hartel [12] using the equation:

% Overrun = [(Wt. of mix − Wt. of same vol. of ice cream)/Wt. of same vol. of ice cream] × 100.

Determination of texture properties was performed by a cycle test of two bytes with the use of texture analyzer model Shimatzu EZ test SX series (Shimatzu, Kyoto, Japan). A 5 mm-diameter stainless steel cylindrical probe with a penetration speed of 2 mm/s was used and analysis included the measurement of hardness (maximum peak force of the first compression cycle during the penetration of the sample, in N) and stickiness (the negative force during withdrawal for the first bite, in N) [33]. All samples were in similar plastic cups, the temperature during analyses was −18 °C and the room temperature was 21 °C.

Color measurements were performed using a portable colorimeter (Lovibond^®^ Colour Measurement LC 100, Tintometer GmbH, Dortmund, Germany). The parameters L* (lightness), a* (redness/greenness), and b* (yellowness/blueness) were measured.

The particle size distribution of the YIC mix and the molten YIC was determined by laser diffraction in a SALD-2300 Shimadzu (Shimadzu Corporation, Tokyo, Japan). After dispersion of the sample in distilled water at 25 °C, a laser beam was transmitted, and the measurement of particle size distribution was based on the angle and the intensity of the scattered light. A refractive index of 1.45 was used for the dispersed phase and the volume percentage was used for the measurement of distribution. The volume percentage of 10–100 μm was used to reflect the degree of fat destabilization [12]. Measurements were performed in triplicate.

Determination of the melting behavior was based on the method described by Sofjan and Hartel [34] with some modifications. Briefly, 70 g of YIC sample with temperature −18 °C and dimensions 4 cm × 5 cm × 6 cm was placed on a 2 mm stainless-steel screen over a funnel in a volumetric cylinder placed on a balance to collect and weigh the melt. Measurement of the time of melting began when the first drop of melt touched the bottom of the cylinder. Weights were recorded every 10 min until the melting was complete. Analysis was carried out in duplicate, and the melting behavior was expressed as the weight of the melt as a percentage of the initial weight.

Counts of yogurt starter microorganisms in the YIC at 1 day and 60 days were enumerated according to the IDF standard method [35].

Antioxidant activity in the YIC samples at 1 day and 60 days as well as in the YAW was evaluated by determining the 2,2-diphenyl-1-picrylhydrazyl (DPPH∙) radical scavenging activity in triplicate. Analysis was carried out according to the assay reported by Moschopoulou et al. [36], with some modifications. Briefly, 100 mg of sample or Trolox (0.25 mg/mL in a mixture of methanol–water 4:1) or methanol (control) were mixed with 900 μL of 0.1 mM DPPH∙ in methanol or methanol (blank tests). After vigorous agitation, incubation in the dark for 30 min at room temperature and then centrifugation (9500× *g* for 5 min) took place. The supernatant was filtered through a 0.45 μm syringe filter (PVDF, Whatman, Maidstone, UK), 250 μL of filtrate were transferred in 96-well microplates, and absorbance at 517 nm was recorded on an ELISA TECAN Sunrise A-5082 spectrophotometer (TECAN Austria GmbH, Grödig, Austria). The antioxidant activity was calculated according to the equation: DPPH∙ radical scavenging activity % = [(A517control − A517sample)/A517control] × 100.

Finally, sensory analysis by six panelists—members of the Laboratory of Dairy Research—was performed once to assess the organoleptic characteristics of the YIC samples after 30 d of storage. All samples were coded with three random digit numbers and were served in random order. Assessors were asked to evaluate the intensity of eight attributes of a given YIC sample using a sensory evaluation card with a five-point descriptive scale, taking into consideration that 0 denoted no intensity, 1 denoted slight intensity, 2 denoted little intensity, 3 denoted moderate intensity, 4 denoted high intensity, and 5 denoted very high intensity. The following attributes were assessed: yogurt aroma, acidity, sweetness, fatty taste, taste, sandiness, wateriness, and color.

### 2.6. Statistical Analysis

The obtained data were statistically evaluated using the software Statgraphics (Centurion XVI Manugistics software, Inc., Rockville, MD, USA). A one-way ANOVA was used to compare the differences among means using the Least Significant Difference test (LSD, *p* < 0.05) and a two-way ANOVA was used to determine the interactions between the addition of YAW and lactose hydrolysis (LH).

## 3. Results and Discussion

### 3.1. YIC Mix Composition

The pH and acidity, as well as the levels of fat, protein, lactose, ash, total solids, and some inorganic elements in the YIC mixes (and consequently in the YIC products) are shown in Table 2. In general, the results were in accordance with the composition reported for commercial YIC [11].

In the production of YIC products, incubation of the YIC mix with yogurt cultures before aging and freezing is the direct acidification, whereas mixing of yogurt and ice cream mix at various ratios to achieve the desired pH is the indirect [14]. Olson et al. [37] reported that when mixing yogurt with ice cream mix at different ratios, the pH ranged from 4.55 for the 100% yogurt to 6.77 for the 100% ice cream mix. In the present study, the pH values of the samples ranged from 5.95 ± 0.04% to 6.09 ± 0.05% and were not significantly (*p* > 0.05) affected by the presence of YAW. The pH of yogurt that was used in the formulations was 4.49 ± 0.02, the pH of the ice cream mix base was 6.42 ± 0.03, and the pH of the YAW was 4.47 ± 0.04. Therefore, mixing strained yogurt with ice cream mix base at a ratio of 1:3 (samples A and AH) and replacing some of the yogurt with YAW (samples B, BH, C, and CH) did not decrease the pH to 4.5–4.6. Usually, this happens in products with higher mixing ratios, e.g., 1:1, and lower total solids content, e.g., 17.58% [22], or in products made by direct acidification [38]. pH values from 6.1 to 6.4 for yogurt ice cream with dietary fibers and probiotics have also been reported [26,30]. In contrast, Bullock et al. [39] mixed strained yogurt with pH 3.717 ± 0.048 and ice cream mix at a ratio of 1:4, which resulted in a frozen dessert with pH 4.27 ± 0.026, without, however, giving any information about the ice cream mix, i.e., ingredients and pH.

The acidity value ranged from 0.36 ± 0.01% to 0.48 ± 0.01% and was significantly affected (*p* < 0.05) by both factors, the addition of YAW and the lactose hydrolysis. However, the presence and the ratio of YAW did not positively correlate with acidity, and thus control samples A and AH presented significantly higher (*p* < 0.05) acidity than samples B and BH (12.5% YAW) and C and CH (18.75% YAW). Acidity expresses the buffering capacity of milk serum, and hence of acid whey. Moreover, acidity, apart from the lactic acid fermentation of lactose, depends also on the MSNF content [12]. Given the fact that the lactose and ash content did not differ significantly among the hydrolyzed and non-hydrolyzed samples, the acidity depended on the protein content of the samples, and the lower the protein content, the lower the acidity. The acidity results obtained in this experiment agreed with those reported by other researchers for YIC [21,26].

Fat content ranged from 4.17 ± 0.06% to 4.95 ± 0.09%, protein ranged from 4.90 ± 0.08% to 6.66 ± 0.08%, and lactose ranged from 5.91 ± 0.17% to 5.97 ± 0.21% (non-hydrolyzed samples) and from 0.19 ± 0.01% to 0.40 ± 0.03% (hydrolyzed samples). Furthermore, total sugars ranged from 21.09 ± 0.46% to 21.70 ± 0.50%, ash ranged from 1.06 ± 0.04% to 1.10 ± 0.01%, and total solids ranged from 30.94 ± 0.14% to 33.74 + 0.06%. From the results, it is obvious that the addition of YAW to the mix formulations affected significantly (*p* < 0.05) fat, protein, and total solids contents, and this was attributed to the very low fat and protein content of YAW. The higher the ratio of YAW in the mix, the lower the content of these components in the YIC mix. The protein content was higher than other reported values for frozen plain yogurts or those enriched with specific flavorings; however, the total solids content was similar [21]. The total sugars contents were lower or higher [14,21,40] than those reported by other researchers. Total sugars consist of lactose and added sweeteners, i.e., sucrose, dextrose syrups, etc., and thus the percentage varies a great deal. Lactose hydrolysis affected significantly (*p* < 0.05), as expected, the lactose content. Lactose hydrolysis was about 95–96%, hence the YIC products herein might be classified as low lactose [19]. Ash content was not affected significantly (*p* > 0.05) either by the addition of YAW or by lactose hydrolysis. Among the inorganic elements that were determined, potassium content was significantly (*p* < 0.05) higher in all samples enriched with YAW (hydrolyzed and non-hydrolyzed lactose) than in the control samples. Sodium was also higher in the samples with YAW. YAW is rich in these soluble inorganic elements [2], and it seems that its presence affected the levels of these elements found in the YIC samples.

### 3.2. YIC Mix Physical Characteristics

The measured physical properties of the YIC mixes are presented in Table 3. The freezing point of the ice cream mix is very important because it indicates the freezing characteristics to be expected from the mix [12]. It is known that the freezing point of a liquid depends on the concentration of soluble ingredients. In the YIC mixes, it ranged from −1.715 ± 0.021 to −2.171 ± 0.032 and was significantly (*p* < 0.05) decreased by lactose hydrolysis because of the monosaccharides content [17,18], while it was not affected by the presence of YAW. An inverse relation between lactose hydrolysis and the freezing point of an ice cream mix has been reported, according to which lactose hydrolysis >75% causes a 0.3 °C depression [17]. Moreover, the YIC mix with 25% yogurt fortified with oligosaccharides had a freezing point of −2.34 [40].

Water activity (a_w_) is a characteristic that is related to the shelf life of food products. In this study, the a_w_ of the YIC mixes ranged from 0.9716 ± 0.0015 to 0.9783 ± 0.0011 and was significantly (*p* < 0.05) affected by lactose hydrolysis. Thus, the hydrolyzed AH, BH, and CH mix samples showed a lower a_w_ than the non-hydrolyzed A, B, and C. This might be the result of the higher number of monosaccharide molecules that bound the free water, compared to the number of lactose molecules, since one mole of hydrolyzed lactose gives one mole of glucose and one mole of galactose. Similar a_w_ values for ice cream mix have been reported [41].

The viscosity of the ice cream mix is a critical characteristic because it determines important properties of the final ice cream product. It is affected by the composition of the mix and especially by the stabilizers, as well as the fat and protein content [12,42]. The viscosity of the YIC mixes measured at 50 rpm ranged from 296.00 ± 26.87 to 145.00 ± 5.66 mPa.s and was significantly (*p* < 0.05) decreased in the order of control A, B, and C samples. This result was attributed to the decreased fat and protein contents of the samples in the same order. Probably, the addition of YAW in samples B, BH, C, and CH diluted the fat–liquid emulsion, decreased their stability, and finally decreased their viscosity. Moreover, the data concerning the effect of acidity on the viscosity of ice cream mixes are contradictory. In the indirect acidification of YIC mix, viscosity has been shown to be either negatively correlated, i.e., the higher the acidity, the lower the viscosity [43], or positively, i.e., the higher the acidity, the higher the viscosity [14]. Lactose hydrolysis significantly (*p* < 0.05) decreased the viscosity of control sample AH and sample BH compared to their counterparts A and B. In contrast, other researchers found that lactose hydrolysis increases the viscosity of the ice cream mix [15,17,18].

### 3.3. YIC Physical and Biofunctional Characteristics

Overrun, the percent increase in volume that occurs because of air incorporation during agitation of the mix in the freezer, is the most important trait of an ice cream product because it can contribute to economic benefit since it affects the weight per unit volume of the ice cream. It is affected by the total solids content of the mix and the type of freezer used. Vertical freezers give overruns from 25% to 50%, compared to 50% to 75% for horizontal freezers [12,42]. The overrun of the produced YIC ranged from 42.02 ± 2.62% to 65.52 ± 1.30% (Table 4), and in general, it was typical for the type of freezer used. However, although the control samples A and AH had a higher total solids content, they presented lower overruns. On the other hand, samples A and AH had high viscosity, and thus their lower overrun was attributed to it since ice cream mixes with high viscosities are known to have limited whipping ability [42]. From the obtained results, it is obvious that both the addition of YAW and lactose hydrolysis affected the overrun significantly (*p* < 0.05). YIC samples B and C exhibited higher overruns than control sample A, and those samples with hydrolyzed lactose exhibited higher overruns than the respective non-hydrolyzed samples. In contrast, other researchers have reported decreased overruns in frozen yogurts with hydrolyzed lactose [44] or no effect [15]. Finally, independent of the presence of YAW or lactose hydrolysis, the achieved overruns were higher than the overruns reported for frozen yogurt [14,15,22,23,26,39,43,44,45].

The hardness of ice cream is principally affected by the initial freezing point, and the lower the freezing point, the softer the ice cream is. Total solids, overrun, and type and concentration of stabilizer also affect it. The higher the overrun, the lower the hardness, while the opposite is true for the total solids content [12,42]. The hardness of the YIC was significantly (*p* < 0.05) affected by both factors, the addition of YAW and lactose hydrolysis. It ranged from 19.56 ± 0.49 N to 81.14 ± 9.34 N (Table 4) and it is obvious that by decreasing the total solids content and increasing the overrun of the samples, which was the effect of adding YAW, the hardness decreased. This was also true for stickiness. On the other hand, the effect of lactose hydrolysis in decreasing the hardness and the stickiness can be attributed only to the increase in overrun, since lactose hydrolysis did not affect the total solids contents. A decrease in the hardness of lactose-hydrolyzed ice creams or frozen yogurts has been also found by other researchers [15,17,46]. A significant decrease in the stickiness of lactose-free frozen yogurt [15] and an increase in the stickiness of lactose-free ice cream [17] have been observed.

The color of ice cream is also an important characteristic as it may affect consumer preferences. Lightness (L* parameter) values ranged from 86.82 ± 3.37 to 92.34 ± 0.23 (Table 4). Samples B, BH, C, and CH fortified with YAW were brighter than control samples A and AH. Moreover, the addition of YAW affected significantly (*p* < 0.05) the greenish color (a* parameter), but not the yellowish (b* parameter). a* values ranged from 0.80 ± 0.07 to −1.68 ± 0.32 and b* values ranged from 7.03 ± 0.40 to 7.96 ± 0.25. The YIC products were greener than the control samples, and this might be attributed to their higher overrun, which better reflected the color of the YAW. Other researchers published similar values for the lightness but lower values for the green color and higher values for the yellow color, e.g., −3.05 ± 0.06 for the a* parameter and 13.27 ± 0.82 for the b* parameter of probiotic yogurt ice cream products [27] or of yogurt ice cream made with second cheese whey concentrated by ultrafiltration [45].

**Figure 1:**
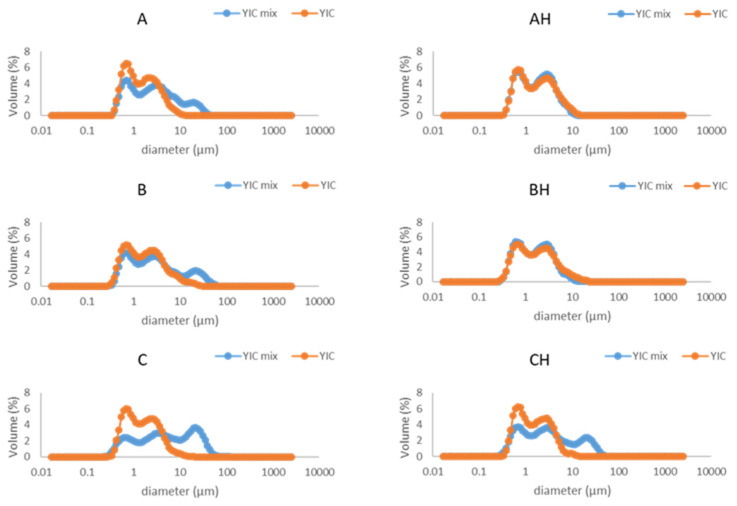
Particle size distribution of yogurt ice cream (YIC) mixes (blue line) and melted yogurt ice cream (YIC) (orange line) made with different levels of added yogurt acid whey (A and AH 0%, B and BH 12.5%, C and CH 18.75%) and lactose hydrolysis (samples A, B, and C 0%, samples AH, BH, and CH > 95%).

Fat destabilization is a critical structural characteristic that affects the shape retention after extrusion and melting rate of the frozen product [12,47]. It indicates the state of dispersion of the fat after freezing, since during freezing fat undergoes partial coalescence induced by the shear forces of agitation and ice crystallization [48]. The particle size distribution of the YIC mix curve was used to compare with the melted YIC curve to determine the extent of fat destabilization (Figure 1). It is obvious that the YICs underwent a low or zero level of fat destabilization, since YIC mixes A, B, C, and CH had larger particles than their corresponding YICs, while YIC mixes AH and BH had particles with almost the same size as the particles in their corresponding YICs. This result might be attributed to the combined effect of the pronounced flocculation of YIC mixes because of low agitation during the mixing of the ice cream base, yogurt, and YAW, and because of the low speed of the dasher during freezing since a domestic vertical ice cream freezer was used.

According to Warren and Hartel [49], increasing the speed of the dasher and the overrun within the freezer increases the shear stress, and hence fat destabilization increases while air cell size decreases. On the other hand, YIC is an ice cream product in which casein flocculation occurs, forming large particles. Particle sizes in yogurts range from 10 to 100 μm, or even higher [50]. To the best of our knowledge, our study is the first to present the particle size distribution in YICs. Moreover, zero fat destabilization in ice creams with whey protein isolate (WPI) as an emulsifier or without an emulsifier has been reported [51,52].

**Figure 2:**
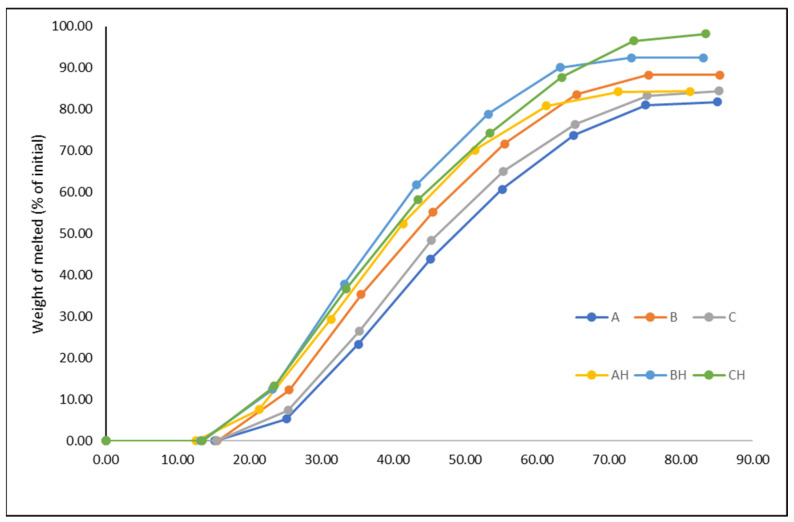
Melting curve of yogurt ice cream made with different levels of added yogurt acid whey (A and AH 0%, B and BH 12.5%, C and CH 18.75%) and lactose hydrolysis (samples A, B, and C 0%, samples AH, BH and CH > 95%).

Melting is another important attribute of ice cream that affects consumers’ preferences. Ice creams with low freezing points tend to melt rapidly, while ice creams with high overrun or fat content tend to melt slowly because air cells act as an insulator and fat stabilizes foam structures [12]. On the other hand, Wu et al. [47] observed that the viscosity of the ice cream mix and fat destabilization significantly affect melting, whereas overrun appears to have an impact only when no stabilizer has been added. The higher viscosity is related to the high melting resistance since it reflects a more stable fat–liquid emulsion. Hence, YIC samples A, B, and C, having higher freezing points and higher viscosity than their corresponding lactose-hydrolyzed samples AH, BH, and CH, melted later (Figure 2). The increased melting rate of lactose-hydrolyzed ice creams has also been reported [17,44]. In contrast, Lindamood et al. [46] found that melting was not affected by lactose hydrolysis.

The biofunctional properties of the YIC samples in terms of the yogurt microorganisms’ counts and the DPPH radical scavenging activity are presented in Table 5. The addition of YAW or lactose hydrolysis did not affect the viable counts of yogurt microorganisms. Populations of *St. thermophilus* were between 7.80 ± 0.34 and 8.45 ± 0.14 log cfu/g at 1 day of storage and remained viable at the same level of numbers for up to 60 days of storage. The viable counts of *L. bulgaricus* ranged from 3.65 ± 0.16 to 3.78 ± 0.07 log cfu/g at 1 day of storage and slightly decreased at 60 days. The total count of yogurt microorganisms was within the legislative limits for YIC, i.e., a minimum of 10^7^ cfu/g [11]. Similarly, yogurt microorganisms or/and probiotic microorganisms were viable at numbers >10^7^ cfu/g for up to 90 days of storage of YICs [22,24,25,26,27].

The DPPH radical scavenging (DPPH RSA) of the YIC samples ranged from 42.1 ± 1.01% to 62.83 ± 1.35% at 1 day and increased at 60 days of storage (Table 5). It was significantly (*p* < 0.05) affected by the presence of YAW and lactose hydrolysis at 1 day, with YIC samples B and BH having significantly higher (*p* < 0.05) values. In general, the antioxidant potential of dairy products is due to conjugated linoleic acid, a-tocopherols, b-carotene, vitamins A and D3, coenzyme Q10 and phospholipids, peptides, proteins, water-soluble vitamins, minerals, and trace elements, and can be changed by processing. Therefore, the DPPH RSA of the YIC samples were from the lipophilic part of the yogurt and the ice cream mix base, especially from the fresh cream used in the formulations, as well as from their hydrophilic part, and especially that from the YAW. Plain set-type bovine yogurt has about 60% DPPH RSA for up to 21 days of storage [36]. The YAW that was used in the YIC formulations had 83.33% DPPH RSA, and it seems that the best formulation for the higher DPPH RSA is that which contained 12.5% YAW.

### 3.4. Sensory Characteristics of YIC

**Figure 3:**
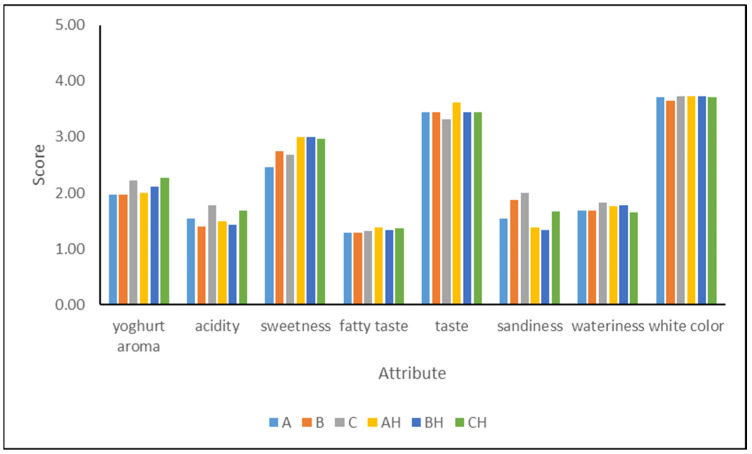
Sensory attributes of yogurt ice cream made with different levels of added yogurt acid whey (A and AH 0%, B and BH 12.5%, C and CH 18.75%) and lactose hydrolysis (samples A, B, and C 0%, samples AH, BH, and CH > 95%).

The results from the sensory evaluation are presented in Figure 3. All the YICs were scored similarly for their taste and white color, and these attributes were from moderate to high intensity. On the other hand, regarding the yogurt aroma and acidity, YICs C and CH fortified with 18.75% YAW presented higher scores than the other YICs, but all the YICs were evaluated as exhibiting from slight to little intensity for these attributes. Acidity was not expected to be very intense since it was not very high (0.36–0.48%). The fatty taste was characterized as exhibiting slight to little intensity, and this was probably due to the relatively low fat content (4.17–4.95%) compared to the fat content of 6% reported by other researchers for such products [14,21,27] as well as to the high sugar content that masks the fatty taste. As expected, sweetness was found to be more intense in lactose-hydrolyzed YICs than in non-hydrolyzed YICs. It is known that lactose hydrolysis increases sweetness and thus a 25% reduction in sugar addition can be made, decreasing, however, the total solids content. Moreover, lactose hydrolysis decreases sandiness and according to Dekker et al. [18] improves the overall acceptability of ice cream. Sandiness in hydrolyzed YICs was scored as less intense than in non-hydrolyzed YICs, but more intense in YICs B and C, which contained YAW 12.5% and 18.75%, respectively. The wateriness of the YIC samples was from slight to little intensity. This might be attributed to the fact that this attribute has been correlated well with protein content, and ice creams with more than 5% protein are not assessed as watery [53].

## 4. Conclusions

The results obtained from this study clearly showed that the acid whey, which is removed during the manufacture of strained yogurt, could be easily incorporated in a formulation of a yogurt ice cream by replacing 50% or 75% of the yogurt. The hardness and stickiness of the products with acid whey significantly decreased while overrun significantly increased, leading to soft scoop products. Moreover, the fortification with acid whey combined with lactose hydrolysis decreases sandiness and increases the overrun even more. The sensory evaluation of the basic attributes did not show major differences among the products. However, in terms of the total solids content, hardness, melting behavior, and biofunctional properties, replacement of yogurt with YAW by 50% (B products) was better than replacement by 75% (C products). In conclusion, the incorporation of YAW in yogurt ice cream seems to be feasible, as it is in the framework of the circular economy and does not leave behind any new waste.

## Figures and Tables

**Table 1:** Formulation (%) of yogurt ice cream mixes made with different levels of added yogurt acid whey.

Materials	A *	B	C	AH	BH	CH
*Ice cream mix base*						
Bovine milk (3.5% fat)	49.17	49.17	49.17	49.17	49.17	49.17
Bovine cream (35% fat)	5.80	5.80	5.80	5.80	5.80	5.80
Bovine SMP-35% Protein	5.93	5.93	5.93	5.93	5.93	5.93
Sucrose	13.50	13.50	13.50	13.50	13.50	13.50
Emulsifiers/Stabilizers	0.60	0.60	0.60	0.60	0.60	0.60
*Strained yogurt (2% fat, 13% MSNF)*	25	12.5	6.25	25	12.5	6.25
*Yogurt Acid Whey*	0	12.5	18.75	0	12.5	18.75
Total	100	100	100	100	100	100

* YAW: yogurt acid whey; LH: lactose hydrolysis; A: 0% YAW and 0% LH; AH 0% YAW and >95% LH; B: 12.5% YAW and 0% LH; BH: 12.5% YAW and >95% LH; C: 18.75% YAW and 0% LH; CH: 18.75% YAW and >95% LH.

**Table 2:** Composition (%), pH, and acidity (%) of yogurt ice cream mixes made with different levels of added yogurt acid whey and lactose hydrolysis (Mean values ± SD).

	A	B	C	AH	BH	CH	YAW:LH *
Total Solids	33.30 ± 0.36 ^a^**	31.72 ± 0.14 ^b^	30.94 ± 0.14 ^c^	33.74 ± 0.06 ^a^	31.92 ± 0.21 ^b^	31.27 ± 0.10 ^c^	NS
Fat	4.84 ± 0.09 ^a^	4.38 ± 0.08 ^b,c^	4.17 ± 0.06 ^d^	4.95 ± 0.09 ^a^	4.44 ± 0.03 ^b^	4.28 ± 0.05 ^c,d^	NS
Protein	6.54 ± 0.08 ^a^	5.43 ± 0.08 ^b^	4.90 ± 0.08 ^c^	6.66 ± 0.08 ^a^	5.66 ± 0.28 ^b^	4.91 ± 0.02 ^c^	NS
Total Sugars	21.15 ± 0.13 ^a^	21.17 ± 0.40 ^a^	21.09 ± 0.46 ^a^	21.42 ± 0.82 ^a^	21.70 ± 0.50 ^a^	21.64 ± 0.31 ^a^	NS
Lactose	5.97 ± 0.21 ^a^	5.82 ± 0.06 ^a^	5.91 ± 0.17 ^a^	0.40 ± 0.03 ^b^	0.25 ± 0.00 ^b^	0.19 ± 0.01 ^b^	NS
Ash	1.10 ± 0.01 ^a^	1.09 ± 0.01 ^a^	1.06 ± 0.06 ^a^	1.09 ± 0.01 ^a^	1.08 ± 0.01 ^a^	1.06 ± 0.04 ^a^	NS
pH	5.97 ± 0.01 ^a,b^	6.06 ± 0.00 ^b,c^	6.06 ± 0.09 ^b,c^	5.95 ± 0.04 ^a^	6.01 ± 0.04 ^a,b,c^	6.09 ± 0.05 ^c^	NS
Acidity	0.48 ± 0.01 ^a^	0.40 ± 0.01 ^c^	0.36 ± 0.01 ^d^	0.46 ± 0.01 ^a^	0.41 ± 0.01 ^b^	0.36 ± 0.01 ^d^	*
*Inorganic elements* (mg/100 g)							
Calcium	154.77 ± 4.95 ^a,b^	155.04 ± 1.52 ^a,b^	151.24 ± 4.10 ^a,b^	153.25 ± 2.13 ^a,b^	156.07 ± 5.15 ^a^	147.36 ± 1.55 ^b^	NS
Magnesium	19.06 ± 0.51 ^a^	19.40 ± 0.57 ^a^	19.02 ± 0.79 ^a^	19.03 ± 0.56 ^a^	19.61 ± 0.80 ^a^	18.71 ± 0.91 ^a^	NS
Potassium	241.07 ± 7.88 ^b,c^	267.05 ± 11.06 ^a^	264.76 ± 9.77 ^a^	229.40 ± 2.85 ^c^	262.11 ± 4.73 ^a^	253.84 ± 2.35 ^a,b^	NS
Sodium	81.94 ± 1.02 ^a^	85.05 ± 3.79 ^a^	86.02 ± 4.63 ^a^	83.52 ± 6.64 ^a^	87.51 ± 2.58 ^a^	86.20 ± 2.49 ^a^	NS

* YAW: yogurt acid whey; LH: lactose hydrolysis; A: 0% YAW and 0% LH; AH 0% YAW and >95% LH; B: 12.5% YAW and 0% LH; BH: 12.5% YAW and >95% LH; C: 18.75% YAW and 0% LH; CH: 18.75% YAW and >95% LH). ** means in the same row with different superscripts differ significantly (*p* < 0.05); YAW:LH interactions between YAW and LH, * significant (*p* < 0.05), NS-not significant (*p* > 0.05).

**Table 3:** Physical properties of yogurt ice cream mixes made with different levels of added yogurt acid whey and lactose hydrolysis (Mean values ± SD).

	A	B	C	AH	BH	CH	YAW:LH *
Freezing point	−1.767 ± 0.013 ^a^**	−1.715 ± 0.021 ^a^	−1.751 ±0.040 ^a^	−2.171 ± 0.032 ^b^	−2.168 ± 0.040 ^b^	−2.153 ± 0.035 ^b^	NS
a_w_	0.9773 ± 0.0023 ^a^	0.9783 ± 0.0011 ^a^	0.9775 ± 0.0008 ^a^	0.9716 ± 0.0015 ^b^	0.9722 ± 0.0008 ^b^	0.9724 ± 0.0003 ^b^	NS
Viscosity (mPa.s)	296.00 ± 26.87 ^a^	189.00 ± 1.41 ^c^	145.00 ± 5.66 ^d^	270.00 ± 14.14 ^b^	146.50 ± 12.02 ^d^	148.00 ± 1.41 ^d^	NS

* YAW: yogurt acid whey; LH: lactose hydrolysis; A: 0% YAW and 0% LH; AH 0% YAW and >95% LH; B: 12.5% YAW and 0% LH; BH: 12.5% YAW and >95% LH; C: 18.75% YAW and 0% LH; CH: 18.75% YAW and >95% LH). ** means in the same row with different superscripts differ significantly (*p* < 0.05); YAW:LH interactions between YAW and LH, NS-not significant (*p* > 0.05).

**Table 4:** Physical properties of yogurt ice cream made with different levels of added yogurt acid whey and lactose hydrolysis (Mean values ± SD).

	A	B	C	AH	BH	CH	YAW:LH *
Overrun (%)	42.02 ± 2.62 ^a^**	51.50 ± 2.36 ^b^	56.86 ± 1.92 ^c^	49.53 ± 2.12 ^b^	58.26 ± 0.09 ^c^	65.52 ± 1.30 ^d^	NS
Hardness (N)	81.14 ± 9.34 ^a^	56.30 ± 2.11 ^b^	43.43 ± 3.91 ^c^	36.07 ± 1.03 ^c^	24.23 ± 1.47 ^d^	19.56 ± 0.49 ^d^	*
Stickiness (N)	−3.32 ± 0.31 ^a^	−1.01 ± 0.10 ^c^	−0.71 ± 0.10 ^c^	−1.45 ± 0.13 ^b^	−0.83 ± 0.08 ^c^	−0.72 ± 0.03 ^c^	*
***Color parameters***							
L*	86.82 ± 3.37 ^a^	90.65 ± 2.34 ^a,b^	92.34 ± 0.23 ^b^	88.92 ± 0.08 ^a,b^	91.26 ± 0.53 ^b^	91.08 ± 1.20 ^a,b^	NS
a*	0.80 ± 0.07 ^a^	−0.69 ± 0.13 ^b^	−1.31 ± 0.08 ^c^	0.54 ± 0.05 ^a^	−0.70 ± 0.00 ^b^	−1.68 ± 0.32 ^d^	NS
b*	7.96 ± 0.25 ^a^	7.44 ± 0.62 ^a^	7.85 ± 0.68 ^a^	7.68 ± 0.46 ^a^	7.30 ± 0.14 ^a^	7.03 ± 0.40 ^a^	NS

* YAW: yogurt acid whey; LH: lactose hydrolysis; A: 0% YAW and 0% LH; AH 0% YAW and >95% LH; B: 12.5% YAW and 0% LH; BH: 12.5% YAW and >95% LH; C: 18.75% YAW and 0% LH; CH: 18.75% YAW and >95% LH). ** means in the same row with different superscripts differ significantly (*p* < 0.05); YAW:LH interactions between YAW and LH, * significant (*p* < 0.05), NS-not significant (*p* > 0.05).

**Table 5:** Microbial counts (log cfu/g) and antioxidant activity of yogurt ice cream made with different levels of added yogurt acid whey and lactose hydrolysis (Mean values ± SD).

	A	B	C	AH	BH	CH	YAW:LH *
*Yogurt starters*							
*St. thermophilus* at 1 d	8.13 ± 0.20 ^a,b^**	8.05 ± 0.19 ^a,b^	7.80 ± 0.34 ^b^	8.45 ± 0.14 ^a^	8.25 ± 0.37 ^a,b^	7.85 ± 0.63 ^a,b^	NS
*St. thermophilus* at 60 d	8.44 ± 0.10 ^a^	8.17 ± 0.09 ^a,b^	7.70 ± 0.20 ^c^	8.19 ± 0.23 ^a,b^	7.96 ± 0.42 ^b,c^	7.87 ± 0.10 ^b,c^	NS
*L. bulgaricus* at 1 d	3.78 ± 0.07 ^a^	3.73 ± 0.02 ^a^	3.65 ± 0.16 ^a^	3.69 ± 0.11 ^a^	3.69 ± 0.08 ^a^	3.68 ± 0.08 ^a^	NS
*L. bulgaricus* at 60 d	3.34 ± 0.36 ^a^	3.27 ± 0.20 ^a^	3.34 ± 0.18 ^a^	3.36 ± 0.36 ^a^	3.31 ± 0.28 ^a^	3.29 ± 0.26 ^a^	NS
*Antioxidant activity*							
DPPH scavenging activity (%) at 1 d	57.67 ± 2.44 ^c,d^	59.96 ± 0.63 ^e^	42.1 ± 11.01 ^a^	48.4 ± 66.09 ^a,b^	62.83 ± 1.35 ^e^	52.47 ± 2.95 ^b,c^	*
DPPH scavenging activity (%) at 60 d	66.26 ± 2.51 ^b,c^	64.27 ± 3.38 ^a,b,c^	65.00 ± 2.12 ^a,b,c^	60.04 ± 4.38 ^a,b^	66.68 ± 3.03 ^c^	59.19 ± 3.76 ^a^	NS

* YAW: yogurt acid whey; LH: lactose hydrolysis; A: 0%YAW and 0%LH; AH 0%YAW and >95%LH; B: 12.5%YAW and 0%LH; BH: 12.5%YAW and >95%LH; C: 18.75%YAW and 0%LH; CH: 18.75%YAW and >95%LH). ** means in the same row with different superscripts differ significantly (*p* < 0.05); YAW:LH interactions between YAW and LH, * significant (*p* < 0.05), NS-not significant (*p* > 0.05).

## Data Availability

The data presented in this study are available on request from the corresponding author.
